# Synchronous cervical lymph node involvement with a papillary thyroid carcinoma and small lymphocytic lymphoma: a case report

**DOI:** 10.1093/jscr/rjaf038

**Published:** 2025-02-05

**Authors:** Sarra Ben Rejeb, Safia Sakly, Amani Hachicha, Kalthoum Dridi, Senda Turki

**Affiliations:** Pathology Department, Security Forces Hospital, Tahar Ben Achour Street, 1050, Marsa, Tunisia; Pathology Department, Security Forces Hospital, Tahar Ben Achour Street, 1050, Marsa, Tunisia; Oto-laryngology Department, Security Forces Hospital, Tahar Ben Achour Street, 1050, Marsa, Tunisia; Laboratory Department, Security Forces Hospital, Tahar Ben Achour Street, 1050, Marsa, Tunisia; Oto-laryngology Department, Security Forces Hospital, Tahar Ben Achour Street, 1050, Marsa, Tunisia

**Keywords:** lymph node, synchronous, lymphoma, papillary thyroid carcinoma

## Abstract

The occurrence of multiple malignancies in the same lymph node is rare, and even more so when these malignancies include both papillary thyroid carcinoma (PTC) metastasis and small lymphocytic lymphoma (SLL). We present a unique case of a 58-year-old male with a history of stable, indolent SLL, who developed metastatic PTC within a lymph node previously involved by lymphoma. Despite initial treatment with total thyroidectomy and cervical lymphadenectomy, post-operative surveillance showed elevated thyroglobulin levels and suspicious lymphadenopathy, prompting further investigation. Fine needle aspiration of the lymph node revealed SLL, but the thyroglobulin level in the aspirate was elevated, suggesting metastasis. A subsequent lymph node dissection confirmed PTC metastasis within the lymphomatous background. This case emphasizes the diagnostic challenges in patients with multiple malignancies, particularly when the presence of an indolent lymphoma complicates the assessment of lymphadenopathy.

## Introduction

The synchronous involvement of a lymph node with a metastatic papillary thyroid carcinoma (PTC) and small B-cell lymphoma (SLL) is uncommon and to the best of the author’s knowledge, only two cases have been reported in the literature [1–4]. We herein described a ‘double trouble’ case of a patient with a known history of SLL who developed a metastasis from PTC occurring in the same lymphomatous lymph node.

## Case presentation

**Figure 1:**
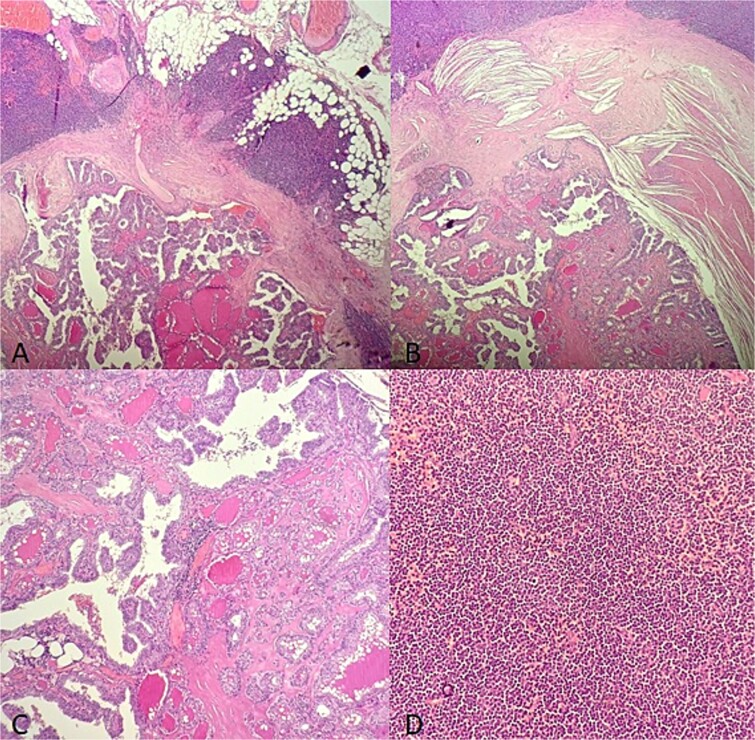
Microscopic findings (A–B): HE X 10: A papillary thyroid carcinoma metastasis into a lymph node involved with a small lymphocytic lympha extending beyond the adipose tissue; (C): HE X 20: Papillary and follicule structure lined with cells showing papillary nuclear atypia; (D): HE X 20: Diffuse small cell lymphoma proliferation.

**Figure 2:**
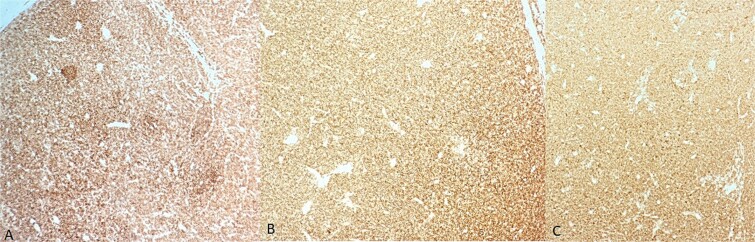
Immunohistochemistry findings. (A) Diffuse and strong membranous staining of lymphomatous cells for CD20; (B) diffuse and strong membranous staining of lymphomatous cells for CD5; (C) diffuse and strong membranous staining of lymphomatous cells for CD23.

A 58-year-old man who was diagnosed with a stable and indolent SLL for 4 years presented with cervical mass enlargement for 7 months. Physical examination revealed a right, 2.5 cm, indurated painless spinal lymph node and a left 2 cm painless jugular cervical lymph node. On cervical ultrasound, multiple suspicious cervical lymph nodes and three thyroid nodules were discovered (8.5 mm EUTIRADS3 in the right lobe and 2 nodules of 5 and 6 mm in the left lobe EUTIRADS4). A fine needle aspiration (FNA) of the left thyroide nodules was performed and concluded to a Bethesda IV follicular neoplasm. Likewise, a FNA of the lymph node was carried out and revealed a monomorphic population of lymphocytes consistent with a SLL. The patient underwent total thyroidectomy with cervical lymphadenectomy. Microscopic examination concluded to multifocal PTC of the left and right lobes. The three nodules were classified pTa with no extra-thyroid extension, vascular or lymph nodes invasion. A 100 m Ci complementary radio-iodine therapy was performed and post-treatment control scintigraphy was negative. The postoperative thyroglobulin levels showed a decrease from 6.8 ng/ml (Day 7) to 1.4 ng/ml (24 months). The follow-up iodine scintigraphy performed at 2 years revealed no specific fixation. However, an increase in thyroglobulin levels (5.8 ng/ml) was observed while anti-thyroglobulin antibody was negative (<6.4 UI/ml). A cervical ultrasound revealed diffuse bilateral lymphadenopathy related to the known hematologic disorder, with lymph nodes in right level IV (20 × 9 mm) and right level VIb (10 × 5 mm) that could be of thyroid origin. A FNA of this lymph node was performed and concluded to SLL. However, the thyroglobulin level in the aspirated fluid was elevated. A cervical lymph node dissection was performed. Microscopic examination revealed lymph node involvement by PTC developed on a lymphomatous background (Fig. 1). The immunohistochemistry analysis showed diffuse and positive staining of the lymphomatous background for CD20, CD23, and CD5 which was consistent with SLL (Fig. 2). The thyroglobulin level decreased post-operatively (0.3 ng/ml). Complementary treatment with 100 mCi radio-iodine therapy was performed. The patient also developed a nasopharyngeal involvement of his SLL and was started on chemotherapy. At 1 year after lymph node dissection, thyroglobulin is negative and the scintigraphy showed no specific fixation.

## Discussion

Herein, we described a challenging and rare case of coexisting SLL and PTC in the same lymph node. It underscores the need for careful diagnostic workup in patients with multiple malignancies. The simultaneous occurrence of thyroid carcinoma and lymphoma in the same patient is very rare. Only two similar cases have been reported in the literature that described the occurrence of both malignancies in the same lymph node. Indeed, in their paper, Hao Z *et al.* described the case of a 75-year-old female patient with concurrent PTC and medullary thyroid carcinoma in different lobes of the thyroid. Additionally, this patient had SLL involvement and PTC metastasis in the same lymph node [4]. Similarly, Sezer *et al.* reported a case of 63-year-old Turkish man with a history of CLL who had SLL involvement and PTC metastasis in the same lymph node [2]. The coexistence of these two distinct malignancies within the same lymph node emphasizes the complexity of interpreting lymphadenopathy in patients with a history of cancer, in particular an indolent lymphoma. In this reported case, the diagnosis was quite difficult because the patient already had enlarged lymph nodes related to his lymphoma, which made the clinical monitoring of lymphadenopathy challenging. Indeed, clinically, an increase in the size of a lymph node could be in this case related to his hematologic disease rather a metastasis from his PTC. Especially since it was a common variant of PTC, with small size and no extra-thyroid extension (pTa), vascular or lymph node invasion initially. Similarly, in this reported case, the iodine scintigraphy showed limited contribution. However, it should be noted that although Iodine-scintigraphy is a valuable tool for detecting thyroid cancer recurrence or metastasis, it can yield false-negative results. They may occur due to tumor dedifferentiation or adjacent activity from other metastases such as lymphomas [5]. Likewise, the small metastases, especially those located deep within the lymphatic chains, may not be detected by scintigraphy due to the limited resolution of the imaging technique. The sensitivity of iodine scintigraphy is generally lower for detecting small or micrometastases compared to other imaging techniques like ultrasound or CT-scans. These findings underscore the importance of using multiple imaging techniques for accurate diagnosis and management of thyroid cancer [6]. In this case, the cervical ultrasound was more sensitive and allowed for the distinction of the lymphomatous lymph nodes, identifying the one suspected of metastatic PTC. As for the FNA, it was of limited contribution for detecting the metastasis. This may be explained by the small size of the metastasis and the presence of diffuse lymphomatous proliferation. However, the increase of thyroglobulin serum and FNA levels was of great diagnostic value and it prompted the clinician's persistent efforts to search for a metastasis. Indeed, it has been reported that thyroglobulin measurement in FNA specimens from lymph nodes is a valuable tool for detecting recurrence of PTC. This method showed higher sensitivity (93.7%) compared to cytology (56.2%) and is useful even in the presence of serum thyroglobulin antibodies [7]. Even more, thyroglobulin measurement in FNA specimens is particularly useful when cytology is inadequate or suspicious, as this case, where the cytology was suggestive of lymphoma.

The etiopathogenesis of this unusual association within the same lymph node remains poorly understood. It is not clear whether it a simple coincidence, or the tumor-induced tumor theory which suggests that 1 tumor triggers changes in the organ micro environment that lead to the development of a second tumor and making the lymphomatous lymph node more prone to tumor homing phenomena [4]. The treatment of these two cancers is independent [2, 4]. In our case, the patient underwent Iodine therapy. As for the treatment of SLL, it depends on the disease progression [8]. In the present case, considering the development of nasopharyngeal involvement with SLL, a chemotherapy was indicated.

## Conclusion

This case report highlighted the rare and complex occurrence of SLL and PTC metastasis within the same lymph node. It underscores the challenges in diagnosing and managing patients with multiple malignancies, particularly in the presence of an indolent lymphoma, which can obscure the identification of metastases. Clinicians should be aware of the possibility of simultaneous malignancies within the same lymph node, and a multi-disciplinary approach is essential for effective management and treatment.
